# Hybrid Constraint-Aware Genetic Optimization for Structurally Coupled Resource Allocation, User Scheduling, and Grouping: An SBMA Case Study

**DOI:** 10.3390/s26206586

**Published:** 2026-10-17

**Authors:** Xiangyu Yang, Jianjian Wu, Jun Wang

**Affiliations:** 1 School of Science and Engineering, Anhui University of Science and Technology, Huainan 232001, China; 2 State Key Laboratory of Digital Intelligent Technology for Unmanned Coal Mining, Anhui University of Science and Technology, Huainan 232001, China; 3 School of Computer Science and Information Engineering, Hefei University of Technology, Hefei 230601, China; 4 School of Mechanical Engineering, Quzhou University, Quzhou 324000, China

**Keywords:** structural coupling, SBMA, sparse code multiple access, blind interference alignment, resource allocation, user scheduling, genetic algorithm, quality of service

## Abstract

This paper studies joint antenna allocation, subcarrier allocation, user activation, and superuser grouping in sparse-code-and-blind-interference-alignment-based multiple access (SBMA), where these decisions also determine the feasible transmission configuration. We formulate the problem as a constrained integer optimization and evaluate a hybrid constraint-aware genetic algorithm (HCAGA) using SBMA as a case study. HCAGA combines a compact integer encoding, feasibility-first selection, uniform crossover, multi-mode mutation, hierarchical structural and rate repair, and periodic QoS-aware local search. Its objective is to maximize the number of users whose rate meets a prescribed threshold. In 50 Monte Carlo realizations with 20 transmit antennas, 5 clusters, 20 subcarriers, and 20 candidate users per cluster, HCAGA serves 54.70 users at 10 dB and 93.54 at 20 dB on average, compared with 48.10 and 85.52 for a Standard GA and 25.00 for the tested budgeted random-search (BRS) implementation. The gain is operating-region dependent: Standard GA is significantly better at 6 dB, whereas the 4 dB difference is not significant and HCAGA is higher at most other tested SNRs. Paired ablations show positive average contributions from hierarchical repair and local search, with repair gains remaining statistically detectable at the tested 6, 12, and 18 dB points. HCAGA also requires substantially more rate evaluations; equal-evaluation-budget performance has not been established.

## 1. Introduction

Wireless networks increasingly operate in regimes in which many users compete for shared time–frequency, spatial, and code-domain resources. Resource allocation, user scheduling, and user grouping are therefore central combinatorial decisions that must balance spectral efficiency, interference, and quality-of-service (QoS) requirements. Non-orthogonal multiple access (NOMA) is one important response to this pressure because it allows multiple users to share the same resource and shifts part of the multiuser separation burden to interference management and decoding [1]. Systematic reviews of 5G resource allocation likewise show that assignment, association, scheduling, spectrum, and power variables are repeatedly coupled in practical multiuser designs [2].

Recent studies demonstrate that several resource variables can indeed be optimized jointly, but they generally do so within a predefined access and transceiver architecture. For example, QoS-aware downlink NOMA resource allocation optimizes transmission decisions under a prescribed multi-antenna NOMA model [3], while secure mmWave-NOMA jointly considers user grouping and power optimization but decomposes the problem into grouping/beam-selection and continuous power/beamforming stages [4]. In SCMA systems, user clustering and downlink beamforming are designed for a fixed MIMO-SCMA C-RAN structure [5], and graph-based resource assignment jointly targets rate and fairness without changing the basic SCMA signal representation [6]. Other studies jointly optimize codebook or resource assignment with bandwidth, power, or QoS variables in SCMA-V2X and RIS-assisted SCMA networks [7,8,9]. Recent work also jointly designs beamforming and resource allocation for STAR-IRS-aided SCMA ISAC systems [10]. Near-global resource-allocation work on uplink SCMA likewise treats resource-element assignment and power coefficients as an MINLP while preserving the underlying SCMA representation [11]. These studies establish the importance of joint optimization, but their resource variables remain embedded in a specified signal representation and transmission architecture.

This distinction can be viewed as the difference between parameter-level coupling and structural coupling. In parameter-level coupling, several variables affect a common objective and are optimized jointly or iteratively, while the underlying access format, signal representation, and transceiver dimensions are fixed in advance. This pattern is explicit in multi-carrier NOMA, where subcarrier assignment and power allocation are treated as iterative subtasks [12]; in NOMA-enabled coordinated multipoint transmission, where base-station clustering and NOMA grouping are alternated with power control [13]; and in virtual Fog-RAN design, where radio-resource allocation and beamforming are solved for a predetermined network architecture [14]. Discrete time-domain allocation also affects LTE-U/Wi-Fi coexistence under fixed-duty-cycle constraints [15]. Hierarchical decoupling with an adaptive genetic algorithm has been applied to PCB placement scheduling [16], while constraint-aware genetic search has been studied for sensor-network coverage [17]. These works address different models and are not direct baselines for SBMA. They show that decomposition and constraint-aware search are both useful design choices; which is preferable depends on the dependencies in the target problem.

Sparse code multiple access (SCMA) and blind interference alignment (BIA) provide complementary mechanisms for constructing such a case. SCMA maps user data to sparse multidimensional codewords, so factor-graph connectivity and codebook geometry influence resource occupancy, interference, and decoding complexity [18,19]. BIA uses signal-space patterns and reconfigurable transmission structures to obtain alignment without relying on instantaneous transmitter channel knowledge [20,21]. Their combination in SBMA creates structural coupling: allocation and grouping variables determine the number and occupancy of superusers, which affect the structural conditions, BIA pattern, supersymbol length, and rates. Antenna and subcarrier assignments also determine the effective SCMA resource association and available signal dimensions. Thus, a change in one decision can change the feasible transmission configuration and the rates of scheduled users.

The SBMA optimization problem is a constrained integer search with heterogeneous decisions, structural feasibility requirements, and user-level QoS constraints. Standard crossover and mutation can create invalid group occupancies or violate minimum antenna, subcarrier, and superuser conditions. A penalty-only search may spend evaluations on candidates that do not represent a valid SBMA configuration. Alternating subproblems also require renewed coordination when a resource or grouping change alters the structure used by the rate calculation. These properties motivate a representation and repair sequence designed for this SBMA model, while leaving the computational trade-off to be measured rather than assumed.

We address two questions: how can the number of users meeting $R_{\min}$ be maximized when SBMA resource and grouping decisions also determine the feasible transmission configuration, and in which tested operating regions does a structure-aware genetic search improve on a generic GA? We propose HCAGA and evaluate it as an SBMA case study. HCAGA searches a compact integer chromosome for joint antenna allocation, subcarrier allocation, user activation, and superuser grouping; each candidate is decoded into the effective SCMA resource association and BIA configuration before fitness evaluation. The objective is to maximize the number of active users satisfying $R_{n,k}\geq R_{\min}$. The contributions are:

- A compact integer chromosome for joint antenna allocation, subcarrier allocation, user activation, and superuser grouping, with the corresponding SCMA resource association and BIA configuration evaluated for each candidate;
- A genetic search with feasibility-first tournament selection and a repair sequence tailored to the structural requirements of SBMA/BIA;
- A hierarchical repair and periodic QoS-aware local search that refines infeasible or low-quality candidates without changing the primary objective;
- A 50-realization evaluation covering SNR, user density, convergence, ablation, threshold, and frequency conditions.

The general design ideas examined here are joint representation of coupled decisions, feasibility-first ranking, and structural repair before QoS-driven refinement. The SBMA/BIA constraints and rate evaluator remain specific to this case study; the experiments do not establish that HCAGA is generally superior for other structurally coupled problems. The remainder of this paper is organized as follows. Section 2 presents the system model and integer formulation. Section 3 describes HCAGA. Section 4 gives the simulation configuration. Section 5 discusses the results and limitations, and Section 6 concludes the paper.

## 2. System Model and Problem Formulation

### 2.1. SBMA Principle

Sparse-code-and-blind-interference-alignment-based multiple access (SBMA) combines SCMA, a code-domain non-orthogonal multiple access technique, with BIA. Within cluster *n*, the scheduled physical users are organized into $C_{n}$ superusers. A superuser may contain one or more users. In the rate abstraction used here, users assigned to a superuser receive an equal average share of its source-coded data/codewords; the detailed assignment of individual users to SCMA streams is not optimized or otherwise modeled.

The transmitter first performs SCMA encoding. Let $b_{n,c\ell }^{\left(t\right)}$ denote the binary input carried by active stream *ℓ* of superuser *c* on the *t*-th antenna allocated to cluster *n*. A prescribed stream codebook $C_{\ell }$ maps the input to a sparse codeword $c_{n,c\ell }^{\left(t\right)}=f_{C_{\ell }}\left(b_{n,c\ell }^{\left(t\right)}\right)$. The active codewords are superposed to form the antenna-wise SCMA symbol vector

(1) $$S_{n,c}^{\left(t\right)}=\sum_{\ell \in L_{n}}c_{n,c\ell }^{\left(t\right)},\;x_{n,c}^{j}={\left[S_{n,c}^{j(1)},\ldots ,S_{n,c}^{j(A_{n})}\right]}^{T}.$$

Here, $L_{n}$ is the active SCMA stream set in the signal-level SBMA description and $S_{n,c}^{j(t)}$ is the *j*-th entry of $S_{n,c}^{\left(t\right)}$. The second expression in Equation (1) is the symbol reconstruction step: entries on the same subcarrier are collected across the $A_{n}$ allocated antennas to form the spatial vector $x_{n,c}^{j}$. This signal-level description does not specify a per-user stream assignment in the optimization model.

The BIA encoder then processes the reconstructed spatial vectors independently on each active subcarrier. Let $G_{n}$ denote the index set of non-empty superusers in cluster *n*. The pattern matrix $V_{n,c}$ extends $x_{n,c}^{j}$ over the supersymbol, and the transmitted extension on subcarrier *j* is

(2) $$X_{n}^{j}=\sum_{c\in G_{n}}V_{n,c}x_{n,c}^{j}.$$

**Figure 1:**
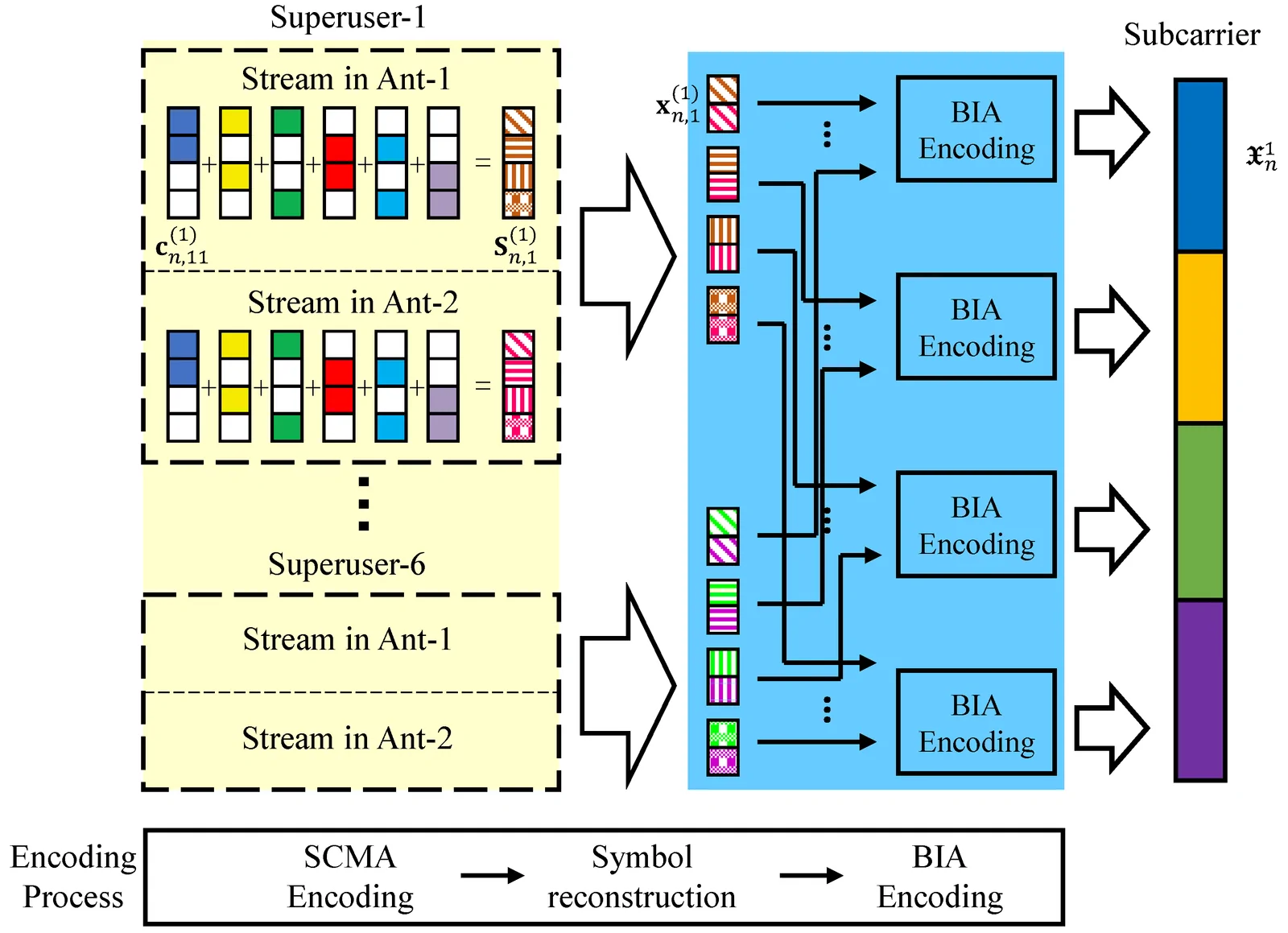
Encoding process of SBMA in cluster *n*, following the SCMA encoding, symbol reconstruction and BIA encoding stages of the original design [22]. Per-antenna binary streams are mapped to SCMA symbols, the *j*-th entries are collected into spatial vectors, and BIA extends the vectors over the supersymbol.

Each user belonging to a given superuser first applies its cancellation matrix to remove interference from the other superusers. The remaining desired signals are then decoded by either a two-stage MPA receiver or a JMPA receiver. The two-stage MPA option uses conventional linear zero-forcing followed by MPA, whereas JMPA jointly detects the desired streams. Figure 1 summarizes the encoding process [22].

In SBMA, antenna and subcarrier assignments determine the effective SCMA resource association, while user activation and grouping determine the number and occupancy of superusers and hence the applicable BIA pattern and supersymbol structure. The codebook constellation values remain prescribed rather than being re-optimized. Thus, resource-allocation and user scheduling/grouping decisions change the effective transmission configuration itself rather than merely selecting locations for fixed transmission blocks.

### 2.2. Optimization Model and Decision Encoding

Consider a downlink SBMA network with *N* clusters, $N_{t}$ transmit antennas, *J* subcarriers, and *U* candidate users per cluster. Let $a=[a_{1},\ldots ,a_{N_{t}}]$ denote the antenna-allocation vector, where $a_{t}\in \left\{1,\ldots ,N\right\}$ assigns transmit antenna *t* to cluster $a_{t}$. Similarly, $s=[s_{1},\ldots ,s_{J}]$ is the subcarrier-allocation vector, where $s_{j}\in \left\{1,\ldots ,N\right\}$ assigns subcarrier *j* to cluster $s_{j}$. For cluster *n*, $u_{n}=\left[g_{n,1},\ldots ,g_{n,U}\right]$ describes user scheduling and grouping. Specifically, $g_{n,k}=0$ means that user *k* is inactive, while $g_{n,k}=c>0$ assigns that user to superuser *c*.

For cluster *n*, the allocated antenna and subcarrier counts are

(3) $$A_{n}=\sum_{t=1}^{N_{t}}I\left(a_{t}=n\right),\;J_{n}=\sum_{j=1}^{J}I\left(s_{j}=n\right),$$

where $A_{n}$ is the number of transmit antennas assigned to cluster *n*, $J_{n}$ is the number of subcarriers assigned to that cluster, and I(·) is one when its argument is true and zero otherwise. Define the assigned-subcarrier set as $J_{n}=\left\{j:s_{j}=n\right\}$, for which $|J_{n}|=J_{n}$. For the rate evaluator, define the antenna-index set $A_{n}=\left\{t\in \left\{1,\ldots ,N_{t}\right\}:a_{t}=n\right\}$, so that $|A_{n}|=A_{n}$.

The occupancy of superuser *c* in cluster *n* and the set and number of non-empty superusers are

(4) $$K_{n,c}=\sum_{k=1}^{U}I\left(g_{n,k}=c\right),\;G_{n}=\left\{c\in \left\{1,\ldots ,U\right\}:K_{n,c}>0\right\},\;C_{n}=\left|G_{n}\right|.$$

Here, $K_{n,c}$ is the superuser occupancy, i.e., the number of active physical users whose grouping label equals *c* in cluster *n*. The label set $G_{n}$ contains only non-empty superusers, and its cardinality $C_{n}$ is therefore the number of active superusers in cluster *n*. This definition counts non-empty groups rather than taking the largest numerical label.

Equation (5) follows by substituting the SBMA received-signal model derived in Section IV of [22] into the established BIA rate derivation in [21]; we use the resulting expression without repeating the receiver derivation. In the MATLAB evaluator, the effective $A_{n}\times A_{n}$ channel for user *k* in cluster *n* on subcarrier *j* is formed as ${\tilde{H}}_{n,k,j}=D_{n}{\left[H_{n,k,j}\right]}_{1:A_{n},A_{n}}$, where $D_{n}=\mathrm{diag}\left(A_{n}^{-1/2},1,\ldots ,1\right)$ implements the first-row scaling used in the code. The BIA dependence in the evaluated expression is represented by the configuration-dependent $C_{n}$ and the supersymbol factor $A_{n}+C_{n}-1$. The implementation uses this resulting rate expression and does not explicitly construct the BIA pattern or cancellation matrices for every candidate; waveform-level receiver validation is outside the scope of this study. For an active user with $c=g_{n,k}>0$, the achievable-rate metric is

(5) $$R_{n,k}=\sum_{j\in J_{n}}\frac{\log\det\;\left(I+\frac{\left(A_{n}+C_{n}-1\right)P_{t}}{A_{n}^{2}C_{n}}{\tilde{H}}_{n,k,j}{\tilde{H}}_{n,k,j}^{H}\right)}{\left(A_{n}+C_{n}-1\right)K_{n,c}},\;c=g_{n,k}>0,$$

where I is the identity matrix of compatible dimension and $P_{t}$ is the transmit-SNR factor under normalized noise power. The factor $A_{n}+C_{n}-1$ accounts for the SBMA supersymbol length. Division by $K_{n,c}$ implements the equal average allocation of source data/codewords among the users assigned to superuser *c*; it does not represent an explicit time-sharing schedule or a modeled stream-level assignment. Accordingly, the optimization imposes no additional $K_{n,c}\leq \left|L_{n}\right|$ constraint. The sum over $j\in J_{n}$ aggregates the rate contributions on the subcarriers assigned to cluster *n*, with one channel use denoting one symbol interval per subcarrier. Because the logarithm is natural, $R_{n,k}$ and $R_{\min}$ are measured in nats per channel use, summed over the subcarriers assigned to the cluster.

The joint optimization problem is formulated as

(6) $$\begin{array}{ccc} \underset{a,s,{\left\{u_{n}\right\}}_{n=1}^{N}}{\mathrm{maximize}}\;\; & K_{s}=\sum_{n=1}^{N}\sum_{k=1}^{U}I\;\left(g_{n,k}>0\;\wedge \;R_{n,k}\geq R_{\min}\right) & \;\\ \mathrm{subject}\mathrm{to}\;\; & a_{t}\in \left\{1,\ldots ,N\right\}, & t=1,\ldots ,N_{t},\\ \; & s_{j}\in \left\{1,\ldots ,N\right\}, & j=1,\ldots ,J,\\ \; & g_{n,k}\in \left\{0,1,\ldots ,U\right\}, & n=1,\ldots ,N,\;k=1,\ldots ,U,\\ \; & A_{n}\geq 2,\;J_{n}\geq 2,\;C_{n}\geq 2, & n=1,\ldots ,N. \end{array}$$

The objective counts an active user as served only when its rate reaches the threshold $R_{\min}$. The three structural constraints ensure that every cluster has sufficient antenna, subcarrier, and non-empty-superuser resources to construct the considered SBMA transmission. In particular, $J_{n}\geq 2$ is the minimum subcarrier-resource condition adopted for the considered SBMA/BIA structure; it is distinct from the dimension of an SCMA codebook. Because all decision variables are integers and grouping changes the superuser occupancies, BIA pattern, and rate normalization, Equation (6) is a constrained combinatorial optimization problem.

A population-based genetic algorithm is adopted because its operators can act directly on the discrete allocation, scheduling, and grouping variables without requiring differentiability or convexity. All coupled decisions are represented in one candidate, which avoids maintaining separate subproblem solutions when an allocation or grouping change alters the transmission configuration. This modeling choice does not imply lower total computational cost: the measured rate-evaluation counts and runtime are reported in Section 5.6.

Accordingly, the physical decisions in Equation (6) are represented by the compact integer chromosome

(7) $$X=[a,s,u_{1},u_{2},\ldots ,u_{N}].$$

For each candidate *X*, the corresponding effective SCMA resource association, BIA pattern, and rates are reconstructed before the objective is evaluated. The prescribed codebook constellation values do not appear as optimization variables. With the dependence on *X* made explicit, the rate and primary objective are denoted by $R_{n,k}\left(X\right)$ and $K_{s}\left(X\right)$, respectively.

For temporary structural violations, we use

(8) $$V_{\mathrm{str}}\left(X\right)=\sum_{n=1}^{N}\max\left(0,2-A_{n}\right)+\sum_{n=1}^{N}\max\left(0,2-J_{n}\right)+\sum_{n=1}^{N}\max\left(0,2-C_{n}\right).$$

Among structurally feasible chromosomes, the tie-breaking fitness is

(9) $$F\left(X\right)=K_{s}\left(X\right)+\epsilon M_{\mathrm{norm}}\left(X\right),\;\epsilon =10^{-3},$$

(10) $$M_{\mathrm{norm}}\left(X\right)=\frac{\sum_{n=1}^{N}\sum_{k=1}^{U}I\left(g_{n,k}>0\right)\max\;\left(0,R_{n,k}\left(X\right)-R_{\min}\right)}{1+\sum_{n=1}^{N}\sum_{k=1}^{U}I\left(g_{n,k}>0\right)R_{n,k}\left(X\right)}.$$

The integer objective therefore remains dominant, while the normalized rate margin breaks ties. After every grouping operation, nonzero superuser labels are canonically relabeled in order of first appearance; this removes equivalent label permutations without changing group membership.

## 3. Hybrid Constraint-Aware Genetic Algorithm

Building on the joint chromosome and fitness model in Section 2.2, this paper proposes a hybrid constraint-aware genetic algorithm (HCAGA) for the structurally coupled SBMA optimization problem. HCAGA combines feasibility-first selection, full-chromosome uniform crossover, four-mode mutation, hierarchical structural and rate repair, and periodic QoS-aware local search. These components maintain or restore the SBMA structural conditions while reevaluating the transmission configuration induced by each resource-allocation or user-grouping change. Relative to a Standard GA, the main design distinction is the SBMA-specific repair and local refinement, not a claim of lower computational cost. BRS provides a low-cost search reference but does not retain information across sampled candidates. Decomposition and alternating optimization can be effective when their subproblems remain manageable; for this model, changing a grouping or resource assignment changes the configuration used by the rate evaluator. HCAGA’s corresponding disadvantages are additional rate evaluations and runtime, more algorithmic settings, and dependence on the SBMA/BIA model. It provides no global-optimality guarantee.

### 3.1. Initialization and Feasibility-First Selection

Each initial chromosome is generated with at least two antennas and two subcarriers per cluster. Every cluster is also initialized with at least two non-empty superusers. For each initialized chromosome, the effective SCMA resource association and BIA-pattern parameters are reconstructed from its decoded assignments before fitness evaluation. The best E=max(1,⌈0.02P⌉) chromosomes are copied to the next generation. The remaining parents are selected by tournaments of size three. A structurally feasible chromosome is always preferred to an infeasible one; among feasible chromosomes, larger F(X) is preferred, while infeasible chromosomes are ordered by increasing $V_{\mathrm{str}}\left(X\right)$. This feasibility-first ordering follows the constraint-handling rules of Deb [23].

### 3.2. Uniform Crossover and Mutation

When crossover is activated with probability $p_{c}=0.8$, every chromosome position independently chooses one of the two parents with probability one half. This full-chromosome uniform operator is used for the antenna, subcarrier, and user-grouping positions. Mutation is activated with probability $p_{m}=0.2$ using four modes: antenna reassignment, subcarrier reassignment, user activation/deactivation, and user regrouping. Their relative weights are (0.15,0.15,0.15,0.20); the implementation normalizes these weights before drawing a mutation mode. Grouping labels are canonicalized after mutation.

After crossover or mutation, the offspring is decoded and its configuration-dependent parameters are updated. Consequently, an apparently local change in a resource or grouping gene can change the effective SCMA resource association, BIA pattern, and dimensions used in the subsequent rate evaluation.

### 3.3. Hierarchical Structural and Rate Repair

The repair sequence is applied after initialization and after every genetic operation. First, antenna deficits are filled by moving antennas from clusters with the largest surplus; the same procedure is then applied to subcarriers. For each cluster, the superuser vector is repaired so that it contains at least two non-empty labels, followed by canonical relabeling. After each structural edit, the effective SCMA resource association and BIA-pattern parameters are updated before rate evaluation. Finally, active users below $R_{\min}$ are processed by rate repair. For a selected user, the repair procedure attempts relocation to another superuser for at most $L_{\mathrm{repair}}$ trials, accepts a non-worsening feasible candidate, and otherwise tries deactivation as a fallback. A final superuser repair and canonical relabeling are then applied. This ordering prevents rate-based edits from invalidating the structural prerequisites of SBMA/BIA while accounting for the induced transmission changes.

### 3.4. QoS-Aware Local Search

Every five generations, local search is applied to the best ⌈0.05P⌉ individuals. Each selected individual receives at most five trials. The neighborhood contains user activation, relocation of a low-margin user, a same-cluster user-label swap, and a superuser split. A candidate is accepted only when it strictly improves the fitness while remaining structurally feasible. Although local search preserves antenna and subcarrier assignments, each grouping move can change the active superuser count and therefore the induced SCMA resource association and BIA pattern; rates are recomputed after every accepted move. The overall pseudocode of the HCAGA algorithm is shown in Algorithm 1.
**Algorithm 1** HCAGA for joint SBMA resource allocation, scheduling/grouping, and induced transmission-configuration optimization.**Require:** $N_{t},N,J,U,R_{\min},P,G_{\max}$ and a channel realization (Section 4)**Ensure:** Best chromosome $X^{*}$ and $K_{s}\left(X^{*}\right)$ under Equations (6) and (9)1:Initialize *P* chromosomes using Equation (7); apply structural repair under Equation (6) and canonical relabeling (Section 3.3)2:Decode each *X* and update its induced SCMA resource association and BIA pattern (Section 2.1 and Section 2.2)3:Evaluate rate, structural violation, and fitness using Equations (5) and (8)–(10)4:**for** g=1 to $G_{\max}$ **do**5:    Copy the elite set to the next population (Section 3.1; Equation (9))6:    **while** the next population is not full **do**7:        Select two parents by feasibility-first tournament selection (Section 3.1; Equations (8)–(10))8:        Apply full-chromosome uniform crossover with probability $p_{c}$ to Equation (7) (Section 3.2)9:        Apply one enabled mutation mode with probability $p_{m}$ (Section 3.2)10:        Apply hierarchical structural/rate repair under Equations (6) and (5), then canonical relabeling (Section 3.3)11:        Update the induced SCMA resource association and BIA pattern (Section 2.1 and Section 2.2)12:        Evaluate the offspring using Equations (5)–(10) and insert it into the next population13:    **end while**14:    **if** *g* is a multiple of the local-search interval **then**15:        Apply QoS-aware local search to the best ⌈0.05P⌉ individuals (Section 3.4; Equations (9) and (10))16:    **end if**17:    Update $X^{*}$ using the feasibility-first ordering (Section 3.1; Equations (6) and (9))18:**end for**19:**return** $X^{*}$

## 4. Simulation Configuration

The baseline simulations use $N_{t}=20$ transmit antennas, N=5 clusters, J=20 subcarriers, $R_{\min}=1.0$ nats per channel use, and U=20 candidate users per cluster. The main SNR sweep covers 0–20 dB in 2 dB increments; the user-density, convergence, ablation, and sensitivity studies use the indicated points at 6, 12, and 18 dB. The density sweep uses U∈{5,10,15,20,25,30}, the threshold sweep uses $R_{\min}\in \left\{0.8,1.0,1.2\right\}$, and the frequency-conditioned channel study uses $f_{c}\in \left\{0.7,1.0,3.0\right\}$ GHz. All simulations were implemented in MATLAB. Every reported scenario uses 50 channel realizations, and algorithms compared within a realization share the same channel sample. All parameters are summarized in Table 1.

We use a transmit-side spatially correlated Rayleigh model for the antenna array; related spatially correlated array settings are considered in [24]. In the simulation matrix representation,

(11) $$H_{n,k,j}=H_{\mathrm{iid},n,k,j}R_{\mathrm{TX}}^{1/2},\;{\left[H_{\mathrm{iid},n,k,j}\right]}_{p,q}∼\mathrm{CN}\left(0,1\right),\;p,q=1,\ldots ,N_{t}.$$

(12) $${\left[R_{\mathrm{TX}}\right]}_{i,j}=\left\{\begin{array}{cc} 1, & i=j,\\ J_{0}\;\left(2\pi d_{ij}/\lambda \right), & i\neq j, \end{array}\right.\;\lambda =\frac{c_{0}}{f_{c}},\;i,j=1,\ldots ,N_{t}.$$

Here, $J_{0}\left(\;\cdot \;\right)$ is the zeroth-order Bessel function of the first kind, $c_{0}$ is the speed of light, and $f_{c}$ is the carrier frequency. In the current implementation, each pairwise separation $d_{ij}$ is drawn independently and uniformly over [0,1] m for each realization; the distance matrix is held fixed across the carrier-frequency conditions within that realization. These distances define the simulation’s correlation construction and are not generated from a geometry-constrained antenna layout. A separate $N_{t}\times N_{t}$ matrix $H_{n,k,j}$ is stored for each cluster, cluster-local user, and subcarrier; its rows represent effective observations/modes, not $N_{t}$ physical receive antennas. Each physical user has one receive antenna. The evaluator selects the first $A_{n}$ rows and the columns indexed by $A_{n}$, then applies the stated first-row scaling to form ${\tilde{H}}_{n,k,j}$.

All full-HCAGA scenarios use the same settings: P=30, $G_{\max}=160$, $p_{c}=0.8$, $p_{m}=0.2$, a minimum of 30 generations, stall patience of 10 generations, $L_{\mathrm{repair}}=2$, and local search every five generations on the best ⌈0.05P⌉ candidates with at most five trials each. Standard GA uses the same compact encoding and nominal population/generation settings, with ordinary structural repair but without hierarchical rate repair or local search. BRS uses a fixed nominal budget of 780 candidate rate evaluations for the cost comparison.

For the main HCAGA–Standard GA comparison, the same realization supplies the channel for both algorithms, and paired two-sided *t*-tests are applied to the per-realization served-user differences at each SNR; Holm correction is applied across the 11 SNR tests. The performance figures report sample means and approximate 95% confidence intervals computed as $1.96s/\sqrt{50}$. The paired ablation uses paired tests and Bonferroni correction across its three SNR points. The main SNR sweep, convergence study, and paired ablation are separate stochastic runs: the convergence and ablation studies share their channel-realization batch and full-HCAGA parameter settings, but use different optimizer random-seed rules, while the main SNR sweep uses its own realization/seed schedule. Their Monte Carlo endpoint means are therefore not expected to be identical; the comparison attributes the former discrepancy to the run/seed schedule rather than to different full-HCAGA parameters.

## 5. Results and Discussion

### 5.1. Served Users Versus SNR

**Figure 2:**
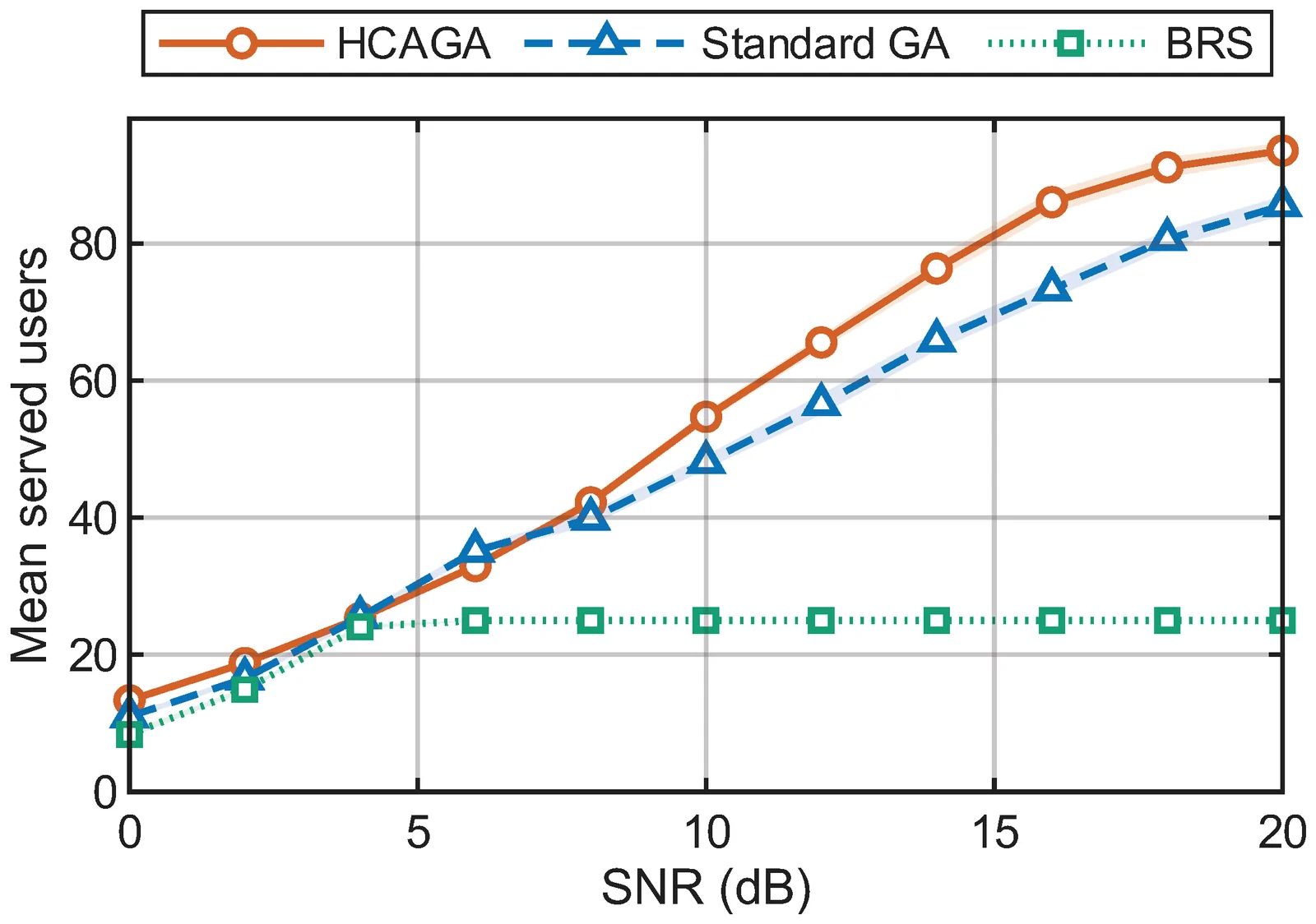
Mean served-user count versus SNR for HCAGA, Standard GA, and BRS (U=20); error bars show approximate 95% confidence intervals over 50 realizations.

Figure 2 reports the served-user mean for U=20 over 0–20 dB. HCAGA serves 54.70 and 93.54 users at 10 and 20 dB, respectively, compared with 48.10 and 85.52 for Standard GA. The comparison is not uniformly favorable to HCAGA. At 4 dB, the HCAGA–GA difference is −0.10 users (paired 95% CI [−0.63,0.43], Holm-adjusted p=0.708), so the difference is not statistically distinguishable. At 6 dB, Standard GA leads by 2.22 users (HCAGA–GA difference −2.22, 95% CI [−2.97,−1.47], adjusted $p=9.34\times 10^{-7}$). HCAGA is higher at most other tested SNRs, supporting a regional rather than universal advantage. A plausible explanation for the low-SNR reversal is that the rate threshold and integer served-user objective create a coarse search landscape when few candidate users can meet QoS; this interpretation is not isolated as a causal mechanism in the experiments.

### 5.2. Candidate-User Density

**Figure 3:**
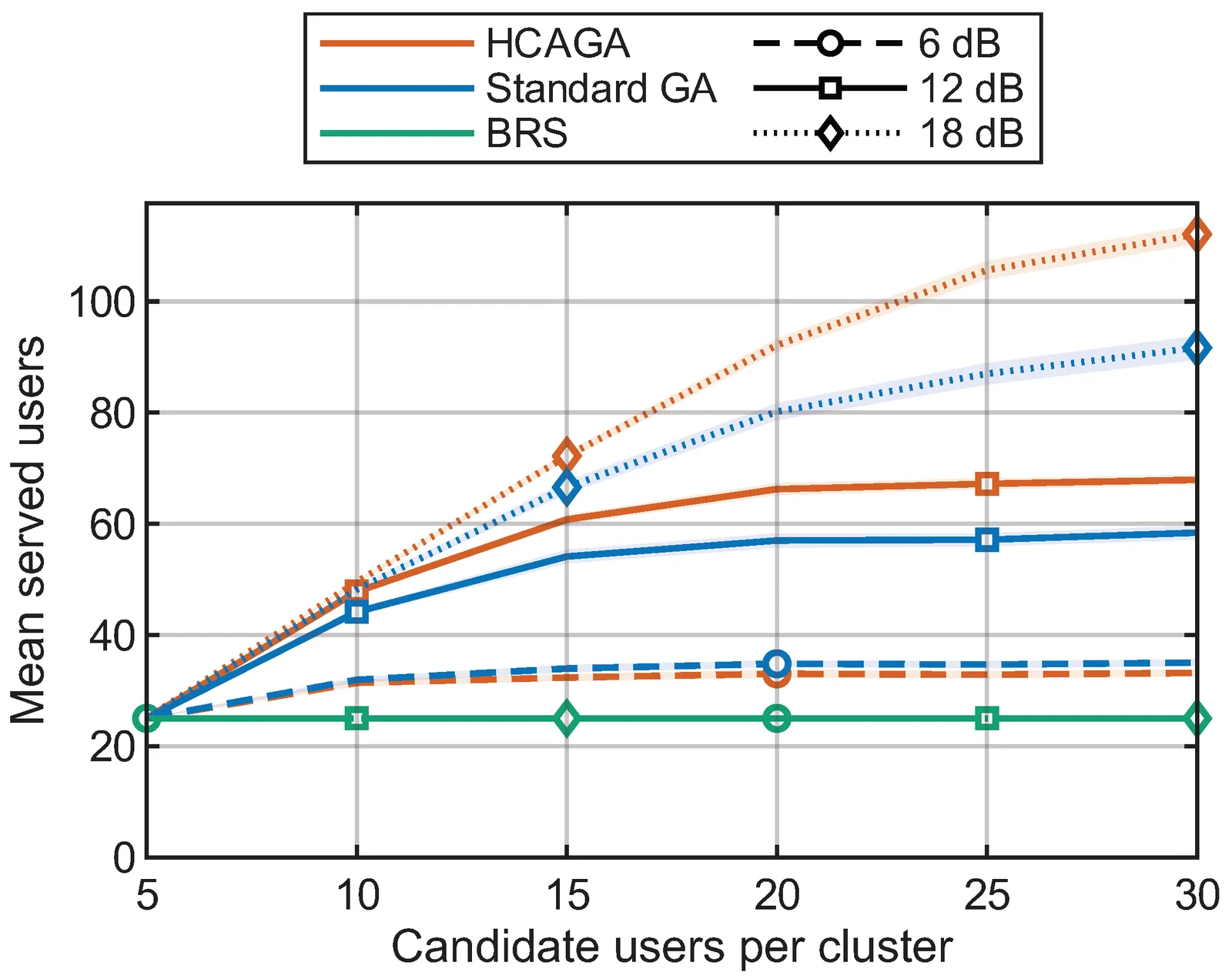
Mean served-user count versus candidate-user density at 6, 12, and 18 dB; error bars show approximate 95% confidence intervals over 50 realizations. Line style encodes SNR and color encodes the algorithm.

Figure 3 examines the number of candidate users per cluster. At U=30, HCAGA serves 33.18, 67.92, and 112.10 users at 6, 12, and 18 dB, respectively; the corresponding Standard GA means are 35.02, 58.40, and 91.68. The density sweep shows that additional candidate users create more scheduling opportunities, particularly at higher SNR, but the HCAGA–GA ranking remains operating-point dependent.

### 5.3. Convergence Behavior

**Figure 4:**
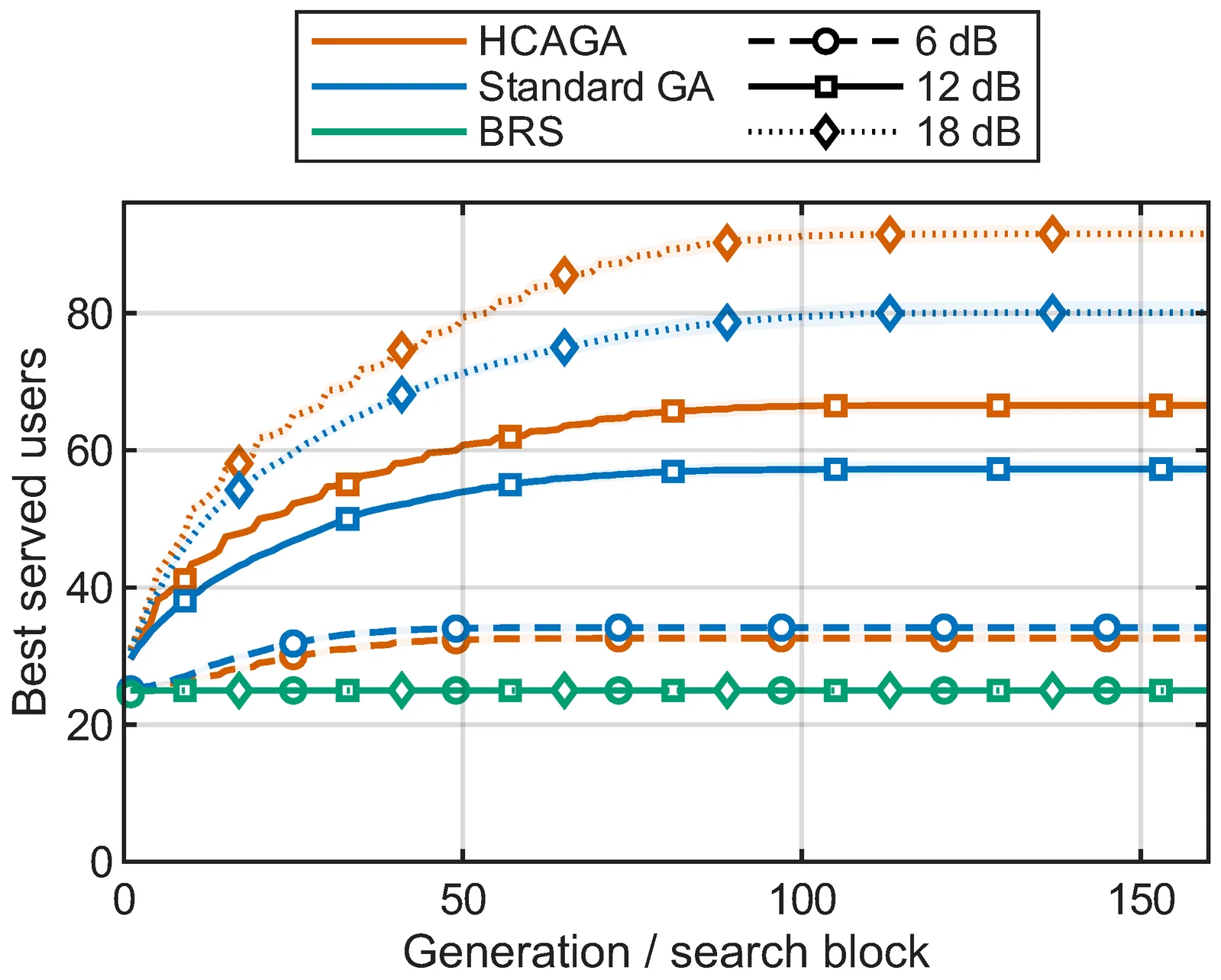
Mean best-so-far served-user count versus generation/search block at 6, 12, and 18 dB; shaded bands show approximate 95% confidence intervals over 50 realizations.

Figure 4 shows best-so-far trajectories at 6, 12, and 18 dB. The HCAGA endpoint means are 32.60, 66.54, and 91.58 users, respectively. The trajectories summarize finite stochastic searches and do not establish universal convergence-rate dominance or the absence of premature convergence.

### 5.4. Paired Ablation of Repair and Local Search

**Figure 5:**
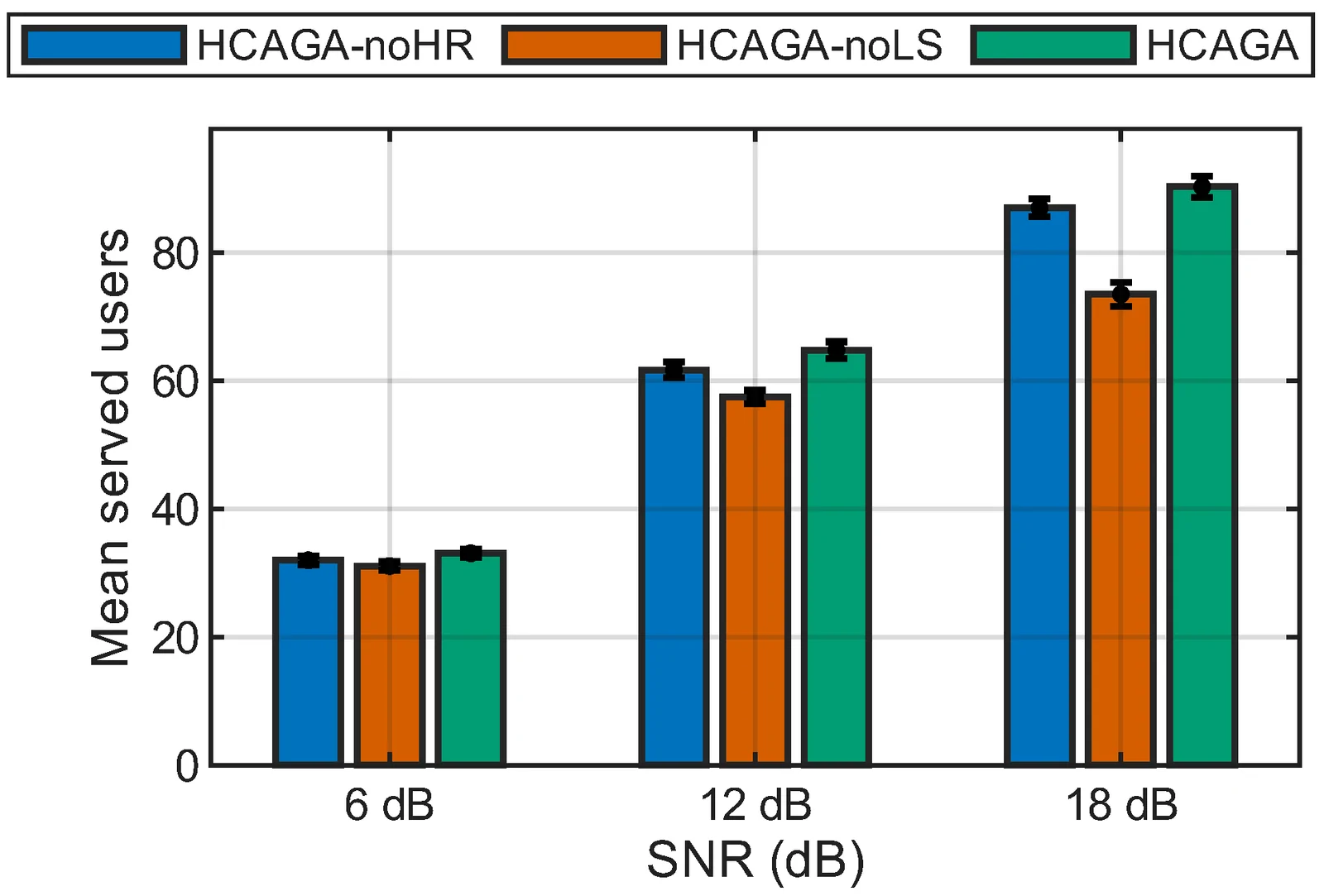
Paired ablation over 50 realizations at 6, 12, and 18 dB; bars show mean served-user count and error bars show approximate 95% confidence intervals. HCAGA-noHR disables rate repair in population evolution and local search; HCAGA-noLS disables local search.

Figure 5 reports a paired ablation over 50 shared channel realizations. At 6, 12, and 18 dB, HCAGA-noHR serves 32.00, 61.72, and 87.02 users; HCAGA-noLS serves 31.10, 57.48, and 73.52; and full HCAGA serves 33.10, 64.80, and 90.30. The full-HCAGA minus noHR paired gains are 1.10 users (95% CI [0.23,1.97], Bonferroni-adjusted p=0.0414), 3.08 ([1.36,4.80], p=0.00222), and 3.28 ([0.87,5.69], p=0.0258), respectively. These results distinguish the HR ablation from the Standard GA comparison: HR has a positive average contribution in the paired ablation, while Standard GA still leads full HCAGA at 6 dB in the main SNR experiment.

### 5.5. Absolute Served-User Gain

**Figure 6:**
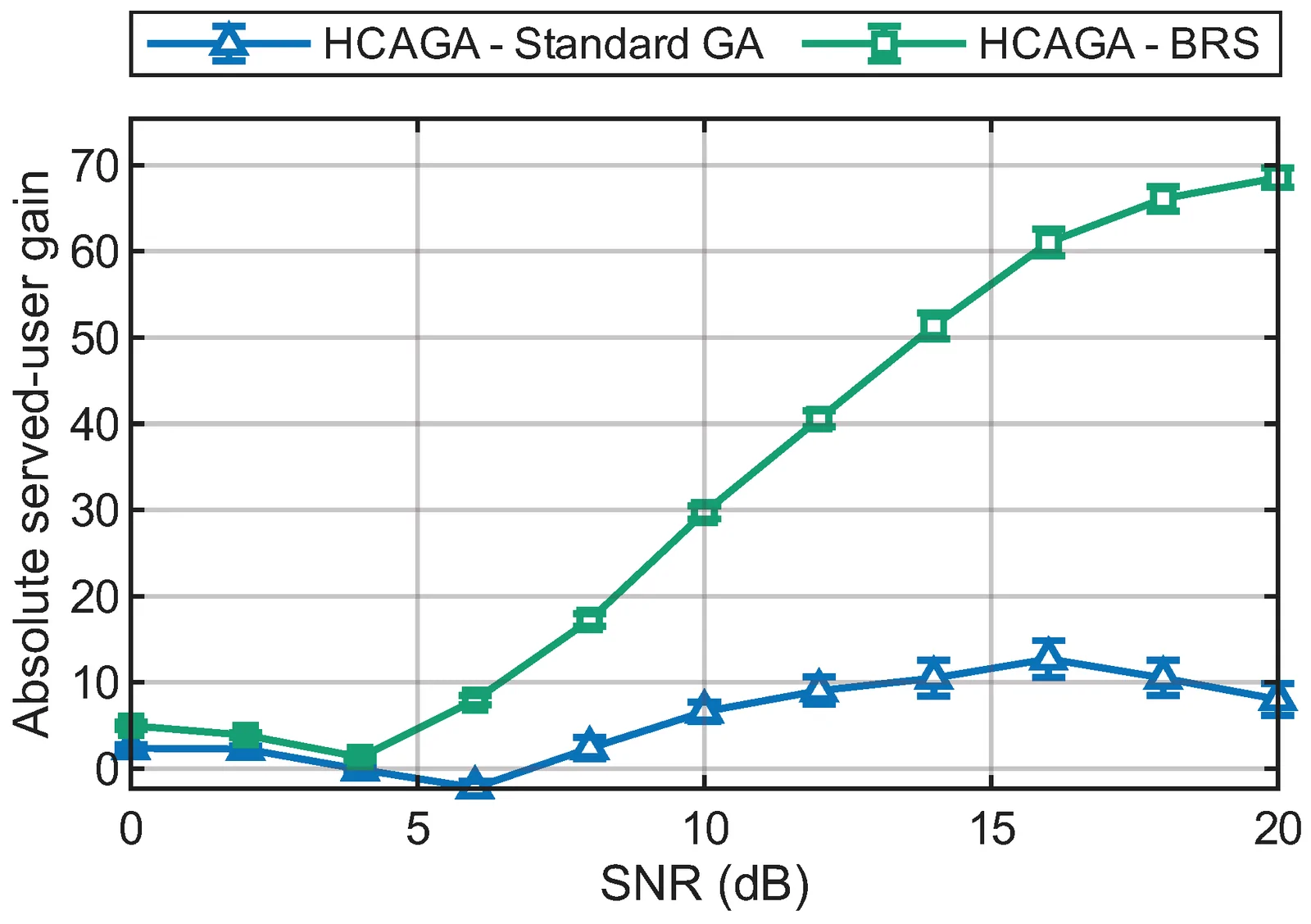
Mean paired absolute served-user gain of HCAGA over Standard GA and BRS; error bars show approximate 95% confidence intervals of the paired differences over 50 realizations.

Figure 6 reports paired absolute served-user differences. HCAGA–Standard GA is −0.10 at 4 dB and −2.22 at 6 dB, but is 12.72 at 16 dB and 8.02 at 20 dB. The HCAGA–BRS difference is 68.54 users at 20 dB. Absolute differences are shown on the user-count scale.

### 5.6. Computational Cost and Limitations

For the baseline setting, the chromosome contains $D=N_{t}+J+NU=140$ integer entries. Before canonical relabeling or constraint pruning, the raw encoding space has cardinality $N^{N_{t}+J}{\left(U+1\right)}^{NU}=5^{40}21^{100}\approx 1.516\times 10^{160}$. This is the unreduced representation count, not the number of feasible or unique transmission configurations. We do not report an analytical feasible-state fraction because satisfying $A_{n}\geq 2$, $J_{n}\geq 2$, and $C_{n}\geq 2$ is jointly determined by allocation and user-grouping decisions, while equivalent group-label permutations make the ratio depend on the encoding representation; the structural constraints are stated explicitly in Equation (6).

For a candidate with $U_{n}^{\mathrm{act}}$ active users in cluster *n*, the configuration-dependent rate evaluator requires approximately $O(\sum_{n}U_{n}^{\mathrm{act}}J_{n}A_{n}^{3})$ work for its $A_{n}\times A_{n}$ matrix log-determinants. A decoding/counting pass is O(D); structural edits and relabeling add overhead. Before caching and early stopping, Standard GA generates $O(P\left(G_{\max}+1\right))$ population candidates. HCAGA also generates rate-repair and local-search candidates. Its measured evaluation count can therefore be written as $E_{\mathrm{pop}}+E_{\mathrm{HR}}+E_{\mathrm{ls}}$, where these terms count population, rate-repair, and local-search candidates evaluated on cache misses. Up to $\left\lfloor G_{\max}/G_{\mathrm{ls}}\right\rfloor E_{\mathrm{ls}}L_{\mathrm{ls}}$ local-search trials are scheduled, and each repair pass can test relocation/deactivation candidates for currently under-threshold users. BRS uses a fixed nominal budget of 780 rate evaluations. We define a rate evaluation as one complete candidate-rate computation on a cache miss; cache hits are recorded separately. Thus, this count is neither the raw evaluator-call count nor the number of individual log-determinants.

**Figure 7:**
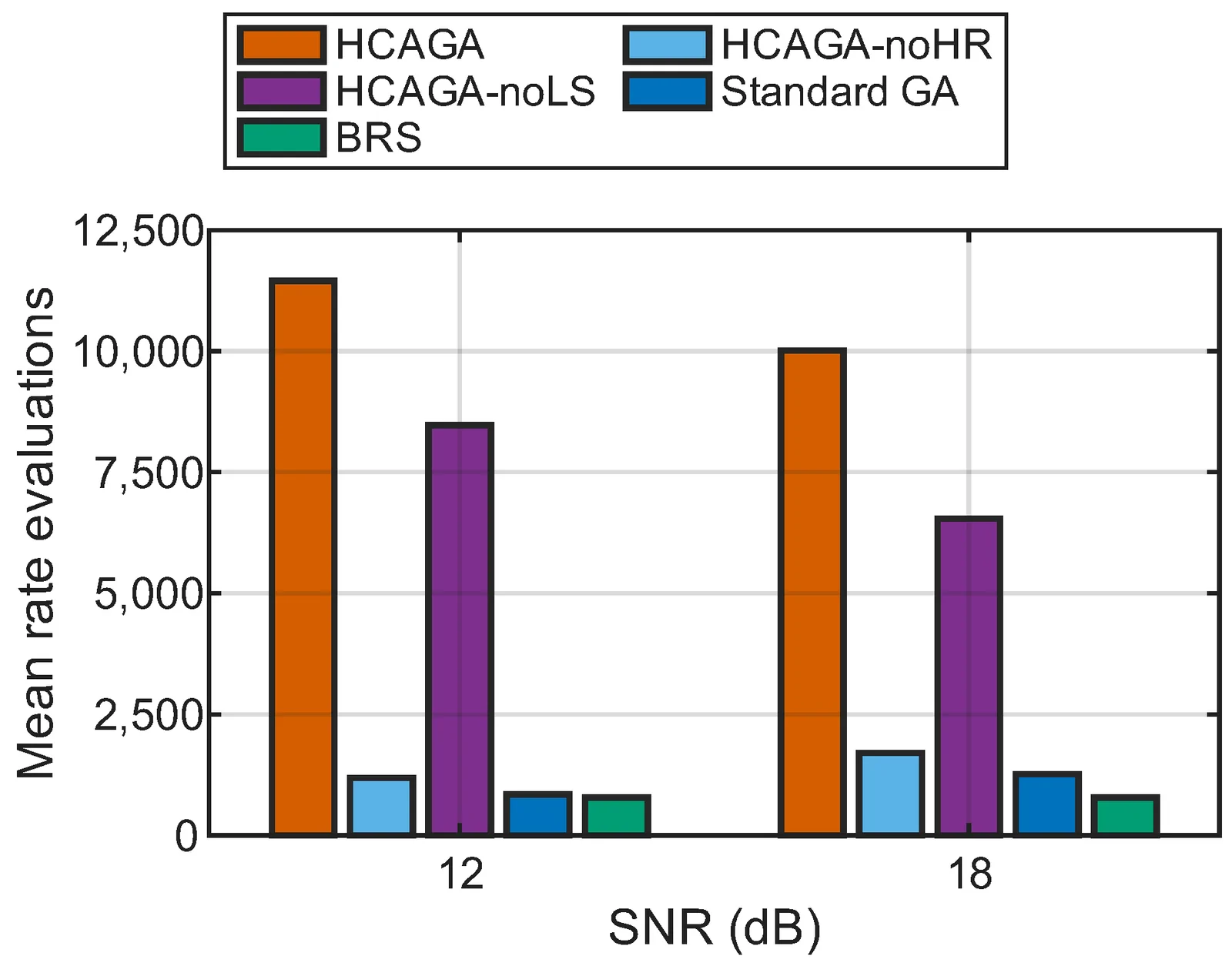
Mean candidate rate-evaluation counts for HCAGA, HCAGA-noHR, HCAGA-noLS, Standard GA, and BRS at U=20, $R_{\min}=1.0$, and 12/18 dB. Each mean uses 50 channel realizations.

Figure 7 compares the measured rate-evaluation cost of HCAGA, HCAGA-noHR, HCAGA-noLS, Standard GA, and BRS at U=20, $R_{\min}=1.0$, and 12/18 dB. The HCAGA, noHR, and noLS means are 11450.40/10012.10, 1188.40/1702.68, and 8472.00/6540.26 evaluations, respectively; Standard GA gives 843.56/1261.48 and BRS uses 780/780. The HCAGA variants use the paired-ablation batch, while Standard GA and BRS use a separate nominal-cost batch with the same system conditions. The corresponding mean wall-clock times are 19.94/23.37 s, 2.29/3.98 s, 13.52/13.40 s, 1.15/2.09 s, and 0.639/0.633 s, respectively. These times describe the recorded MATLAB runs and are not hardware-independent complexity measures.

Together with the paired-ablation results in Figure 5, these costs clarify the trade-off between the HCAGA variants. At 12/18 dB, noHR serves 61.72/87.02 users, compared with 64.80/90.30 for full HCAGA and 57.48/73.52 for noLS. Thus, under the tested settings, noHR retains most of the full method’s served-user performance at substantially lower evaluation cost and can be a practical candidate when computational cost matters. If additional evaluations are acceptable, full HCAGA provides the highest served-user mean in the paired ablation. The present experiments do not separate the effect of the search mechanisms from the effect of their additional evaluations under a common hard budget; equal-budget superiority is not claimed.

The integer primary objective, the small rate-margin tie-break, and strict-improvement local search may cause stagnation on plateaus. The convergence curves do not establish that premature convergence is absent. Larger neighborhoods and adaptive operator control remain possible extensions. The simulations also use one spatially correlated Rayleigh family and finite samples. The model retains prescribed codebook constellations and a configuration-dependent rate evaluator based on Equation (5); detailed user data stream scheduling and waveform-level receiver validation are outside its scope.

### 5.7. QoS-Threshold Sensitivity

**Figure 8:**
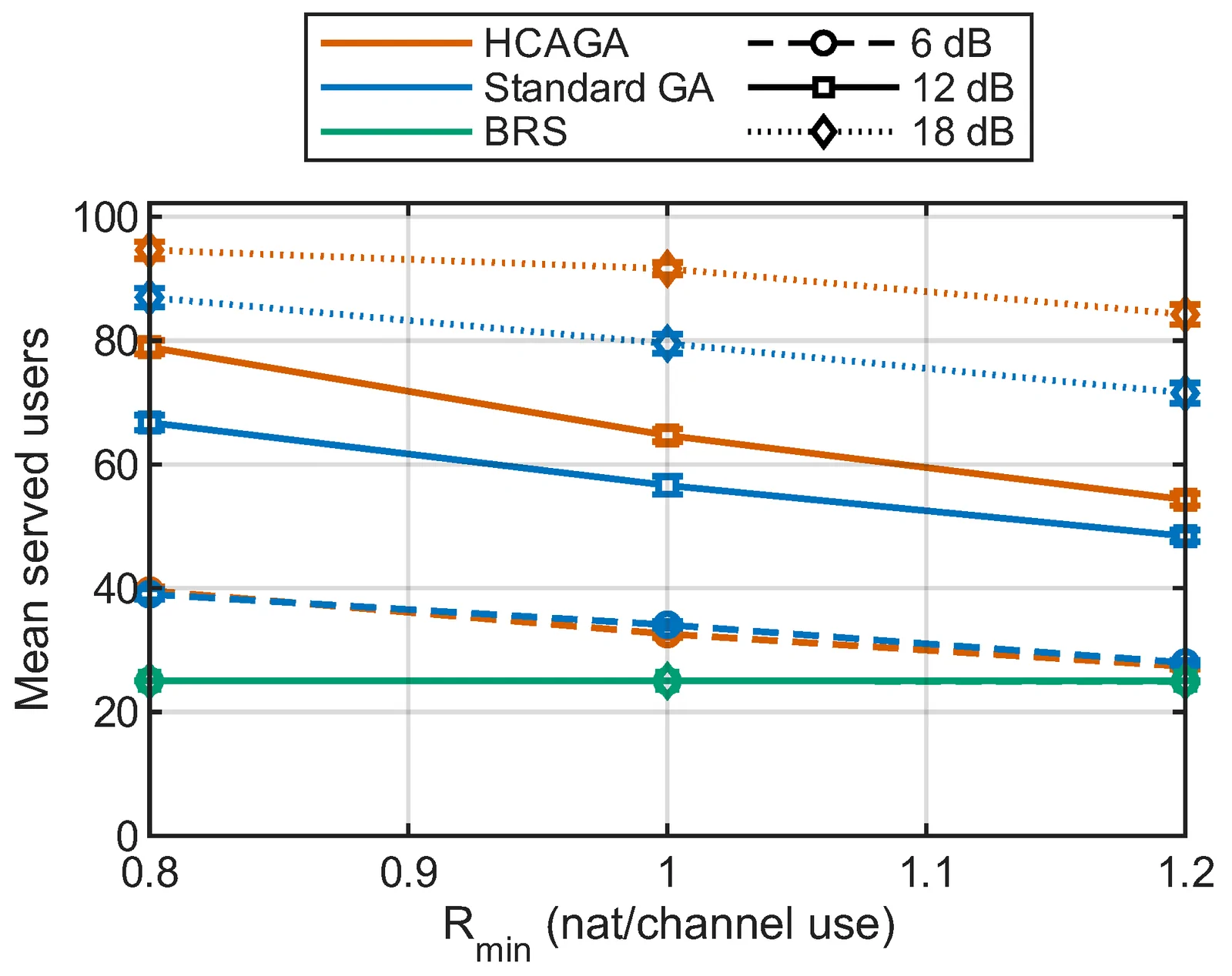
Mean served-user count versus $R_{\min}$ at 6, 12, and 18 dB; error bars show approximate 95% confidence intervals over 50 realizations.

Figure 8 varies $R_{\min}$ over 0.8, 1.0, and 1.2 nats per channel use. At 12 dB, the HCAGA means decrease from 78.94 to 64.68 and 54.30 users as the threshold increases; at 18 dB, they are 94.62, 91.62, and 84.22. The tighter threshold reduces the number of served users, as expected. HCAGA remains higher than Standard GA at the tested medium/high-SNR conditions, but the low-SNR ranking is not uniform across thresholds.

### 5.8. Frequency-Conditioned Channel Study

**Figure 9:**
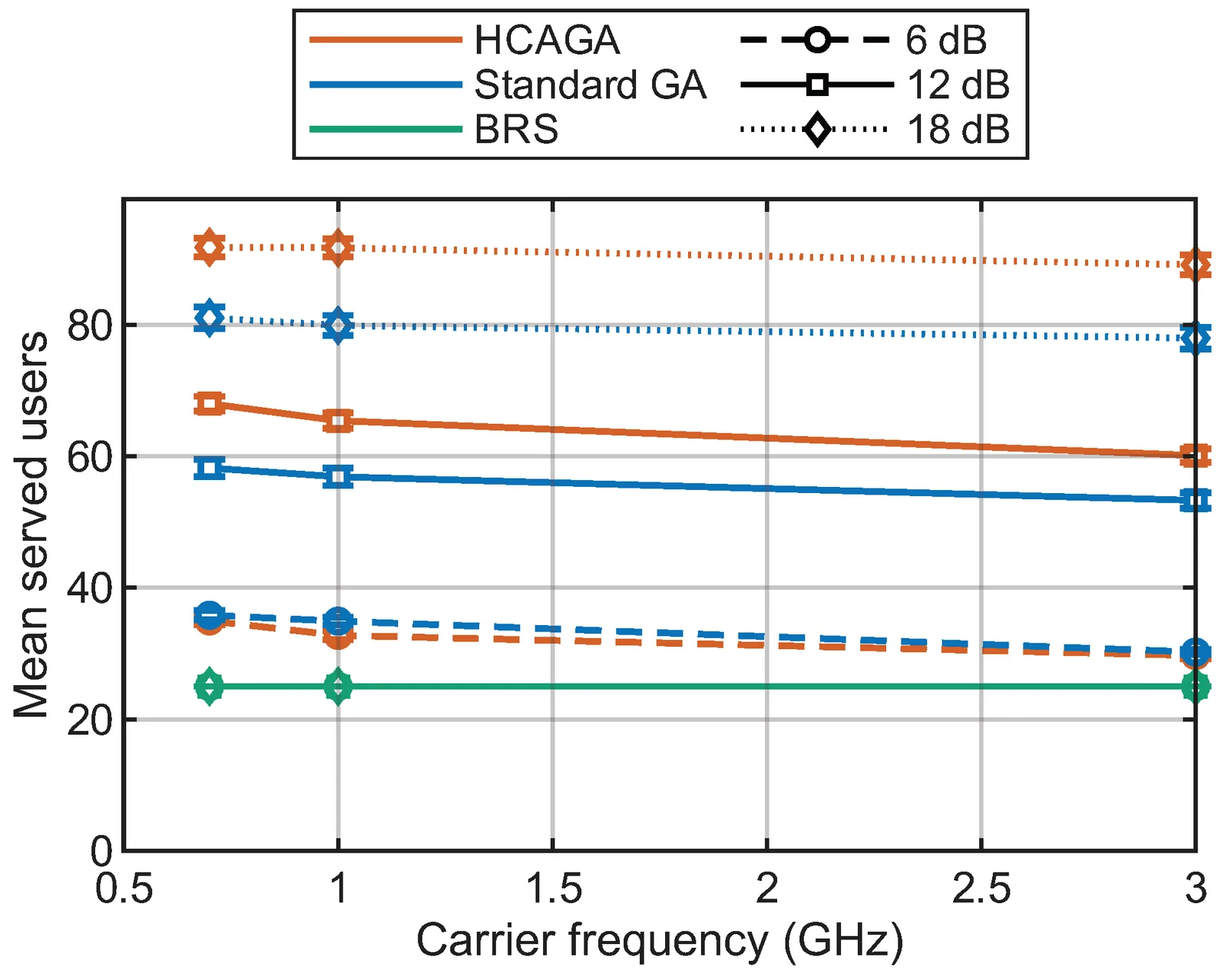
Mean served-user count versus carrier frequency under the same spatially correlated Rayleigh formula; error bars show approximate 95% confidence intervals over 50 realizations.

Figure 9 changes $f_{c}$ in the same correlation model, thereby changing $\lambda =c/f_{c}$ while retaining the realization’s pairwise distance matrix. At 12 dB, the HCAGA means at 0.7, 1.0, and 3.0 GHz are 67.98, 65.40, and 60.10 users; at 18 dB, they are 91.76, 91.68, and 89.10. HCAGA remains above Standard GA at those medium/high-SNR conditions, while small low-SNR reversals persist. This is a frequency-conditioned comparison within the stated spatial-correlation model, not a comparison of different fading families.

## 6. Conclusions

This paper evaluated HCAGA for an SBMA case study in which antenna/subcarrier allocation and user activation/grouping determine the effective SCMA resource association, BIA configuration, supersymbol structure, and rates. Joint encoding, feasibility-first selection, canonical relabeling, hierarchical repair, and local search account for these dependencies. The 50-realization results show higher average served-user counts than Standard GA at most tested medium/high-SNR points, with a significant reversal at 6 dB. Paired ablations show positive average contributions from HR and LS under the tested nominal settings. Threshold and carrier-frequency studies support the regional nature of the gain within the stated model. Rate evaluation results demonstrate the computational complexity of HCAGA, including the proposed hierarchical rate repair and local search mechanisms. HCAGA provides a feasible solution for structurally coupled resource allocation and user pairing problems in SBMA systems. Future work will examine adaptive operator control, fairness-aware objectives, and the general algorithm design for universal problem scenarios.

## Figures and Tables

**Table 1:** Simulation parameters.

Parameter	Value	Description
$N_{t}$	20	Transmit antennas
*N*	5	Clusters
*J*	20	Subcarriers
*U*	20 (5–30 in density sweep)	Candidate users per cluster
$R_{\min}$	1.0	Minimum rate threshold (nats per channel use)
*P*	30	Population size for both GAs
$G_{\max}$	160	Maximum generations/search blocks
$p_{c}$, $p_{m}$	0.8, 0.2	Crossover and mutation probabilities
ϵ	$10^{-3}$	QoS-margin tie-break weight
*E*, $E_{\mathrm{ls}}$	⌈0.02P⌉, ⌈0.05P⌉	Elite and local-search counts
$L_{\mathrm{repair}}$	2	Rate-repair trials
$G_{\mathrm{ls}}$, $L_{\mathrm{ls}}$	5, 5	Local-search interval and trials
Monte Carlo	50	Realizations in each reported scenario
Threshold sweep	{0.8,1.0,1.2}	$R_{\min}$ values (nats per channel use)
Carrier frequency	{0.7,1.0,3.0} GHz	Frequency-conditioned channel study
BRS budget	780	Nominal random-search evaluations

## Data Availability

The raw data supporting the conclusions of this article will be made available by the corresponding author on request.
